# Forecasting knee arthroplasty surgery with deep learning: an integrated approach using routine clinical data and radiographs

**DOI:** 10.1302/2633-1462.77.BJO-2025-0268.R1

**Published:** 2026-07-14

**Authors:** Omar Musbahi, Thomas A. G. Hall, Alexis Alibert, Gareth G. Jones, Justin Cobb, Richard J. van Arkel

**Affiliations:** 1 MSk Lab, Department of Surgery and Cancer, Imperial College London, London, UK; 2 Biomechanics Group, Department of Mechanical Engineering, Imperial College London, London, UK

**Keywords:** Artificial intelligence, Knee, Arthroplasty, Total knee arthroplasty, Osteoarthritis, Prediction, arthroplasty surgery, knee arthroplasty, osteoarthritis (OA), knee, knee osteoarthritis, radiographs, total knee arthroplasty (TKA), knee joint, MRI, partial knee arthroplasty

## Abstract

**Aims:**

We aimed to develop and validate a practical deep learning model integrating commonly collected clinical data and knee radiographs to predict the need for knee arthroplasty (total or partial) in patients with, or at risk of, knee osteoarthritis, as well as the time to surgery.

**Methods:**

Data from the Multicentre Osteoarthritis Study (MOST) and the Osteoarthritis Initiative (OAI) were used. The MOST dataset, comprising 3,026 patients, was the primary training and testing cohort, while the OAI dataset provided external validation. The final architecture was based on DenseNet-201, with a head that combined outputs from the radiological analysis with commonly collected clinical data. Model evaluation used the area under the receiver operating characteristic curve (AUROC) and precision-recall curve (AUPRC).

**Results:**

The integration of clinical and radiological data significantly improved predictive accuracy. The combined model achieved an AUROC of 0.85 and AUPRC of 0.62, outperforming models using either data source alone. External validation with the OAI dataset yielded an AUROC of 0.79, confirming the model’s generalizability. The AUROC and AUPRC for surgical interventions within 40 months was 0.83 and 0.26, respectively, on the validation dataset, demonstrating higher predictive accuracy for earlier surgical needs.

**Conclusion:**

This study highlights the potential of deep learning models, which integrate clinical and radiological data, to predict the need for knee arthroplasty. The robust performance and generalizability of the developed model could streamline clinical pathways and predict local demand for surgery during the next three years. This will facilitate resource planning for providers and accurate and timely access to surgical interventions for patients.

Cite this article: *Bone Jt Open* 2026;7(7):926–933.

## Introduction

Affecting over one-fifth of the global population aged over 40 years,^1,2^ osteoarthritis (OA) is a primary cause of disability, leading to pain, impaired mobility, and a substantial socioeconomic burden.^2-4^ The primary surgical intervention for severe knee OA is knee arthroplasty.^5^ Increasing demand for knee arthroplasty is straining healthcare systems,^6^ and there is need to efficiently identify which patients will require knee arthroplasty surgery and when.

AI is revolutionizing healthcare by providing advanced tools for data analysis, interpretation, and decision-making.^7^ Applying complex statistical models to large datasets, these technologies have the capacity to identify and predict patterns, surpassing human analytical capabilities. In the context of knee OA, previous studies have explored the use of machine learning algorithms for predicting the need for knee arthroplasty based on clinical data and medical images.^8-12^ The findings have highlighted the potential of neural networks and decision trees in the identification of patterns indicative of OA severity and surgical necessity.^9,13^ However, most studies have either focused on single-modality data or been limited by small, homogeneous datasets.^14^ There is a notable gap in the literature studying the integration of diverse data types, such as radiological images and comprehensive patient medical histories, to enhance predictive accuracy. Furthermore, existing models often lack external validation, limiting generalizability and clinical applicability.

The primary objective of this study was to develop and validate a robust deep learning model that combines radiological images with routinely collected patient clinical data to predict the need for knee arthroplasty in individuals with knee OA. Utilizing extensive and diverse datasets, the study aimed to address the limitations of previous research through comprehensive data integration. We hypothesized that this combined model would significantly improve prediction accuracy and provide reliable estimates for the timing of surgical intervention, ultimately aiding surgical planning and reducing the burden on healthcare systems.

## Methods

### Study design and data source

We designed a deep learning predictive model using two prospectively collected longitudinal datasets (Table I). The Multicentre Osteoarthritis Study (MOST) dataset with 3,026 patients was used for training and testing with a patient-level hold-out set.^15^ The Osteoarthritis Initiative (OAI) dataset with 4,796 patients was used for external validation.^16^ Access was granted to the corresponding author for both the MOST and OAI datasets, with the analysis conducted retrospectively. Ethical approval and informed consent were obtained from all participants in both OAI and MOST datasets.^16,17^

**Table I.** Baseline characteristics and knee arthroplasty outcomes for the Multicentre Osteoarthritis Study (MOST) and Osteoarthritis Initiative (OAI) cohorts used for training and testing, respectively.

Variable	MOST	OAI
Patients, n	3,026	4,796
Mean age, yrs (SD)	62.5 (8.1)	61.3 (9.2)
**Sex, n (%)**		
Male	1,206 (39.9)	1,992 (41.5)
Female	1,820 (60.1)	2,804 (58.5)
Mean BMI, kg/m^2^ (SD)	29.4 (5.1)	/A
Mean WOMAC (SD)	20.4 (17.5)	12.0 (15.3)
Family history of OA, n (%)	2,113 (69.8)	N/A
Non-Caucasian, n (%)	502 (16.6)	919 (19.2)
**Outcomes, n (%)**		
Did not undergo surgery	2,531 (83.6)	4,357 (90.8)
Underwent knee arthroplasty	495 (16.4)	439 (9.2)
Before 40 months	236 (7.8)	131 (2.7)
From 40 to 70 months	149 (4.9)	148 (3.1)
After 70 months	110 (3.6)	160 (3.3)
One knee replaced	319 (10.5)	330 (6.9)
Both knees replaced	176 (5.8)	109 (2.3)

N/A, not available; OA, osteoarthritis; WOMAC, Western Ontario and McMaster Universities Osteoarthritis Index.

### Inclusion and exclusion criteria

Data from all patients in the MOST database were included in the training of convolutional neural networks (CNN) and random forest models. Both the OAI and MOST datasets comprise patients either with knee OA or at risk of developing it. The OAI dataset includes patients aged 45 to 79 years at enrolment, while the MOST dataset contains data on those enrolled between 50 to 79 years old.^15,16^

### Outcomes

The models were first trained to predict surgery on a binary prediction of surgical intervention (total or partial knee arthroplasty) at any point during the enrolled study periods. Second, we conducted an exploratory study to predict the timing of surgical interventions across different time bins, identifying patients most in need of surgical intervention. Class 0 was defined as no surgery; Class 1 was surgery before 40 months; Class 2 was 40 to 70 months; and Class 3 was surgery after 70 months. Both studies deployed class weights during training to account for the imbalance between surgery and non-surgery cases.

### Algorithms for clinical data

Initial model testing considered various machine learning algorithms, including random forest, decision trees, support vector machines, gradient descent, logistic regression, and XGBOOST.^18^ Clinically relevant and commonly collected data inputs were selected, namely age, sex, and race as defined in the MOST database, BMI,^19^ Western Ontario and McMaster Universities Osteoarthritis Index (WOMAC) pain score,^20,21^ and family history of OA. These limited inputs were selected for ease of collection during clinical practice. The inclusion of Kellgren-Lawrence (KL) grade was investigated, but it was excluded from the final integrated model, with the goal of developing a fully automated system independent of expert medical assessment.

### Algorithms for radiological data

CNNs were used for analysis of knee radiographs, leveraging transfer learning on weights pretrained on ImageNet (Stanford University, California, USA). An initial study was used to select a base architecture from DenseNet-201,^22^ ResNet-50,^23^ EfficientNetB0,^24^ and Inception V2,^25^ with additional dense layers and dropout layers to prevent overfitting. The best performing architecture was then refined by tuning parameters such as learning and dropout rates.

### Combining clinical and radiological data

We investigated two approaches to integrate predictions from both radiological images and clinical data: 1) weighted contribution, which involved assigning optimized weights to the probabilities of the best-performing radiograph and clinical data models for a new prediction; and 2) combined archiecture, a more advanced approach where clinical data were fed into to the final layer of the neural network, which was then retrained with the rest of the network frozen.

### Performance metrics and explainability

The primary performance metric was the area under the receiver operating characteristic curve (AUROC) for each model, measuring the true positive rate against the false positive rate. Area under the precision-recall curve (AUPRC) was also calculated using the average precision (AP) method. Grad-CAM (gradient-weighted class activation mapping) was used to visualize the areas of the radiographs that influenced the model’s predictions.^26^ Grad-CAM highlighted regions of the knee joint that were influential in the model’s decision-making process, providing insights into the model’s interpretability.

## Results

### Predictions on clinical data

**Fig. 1:**
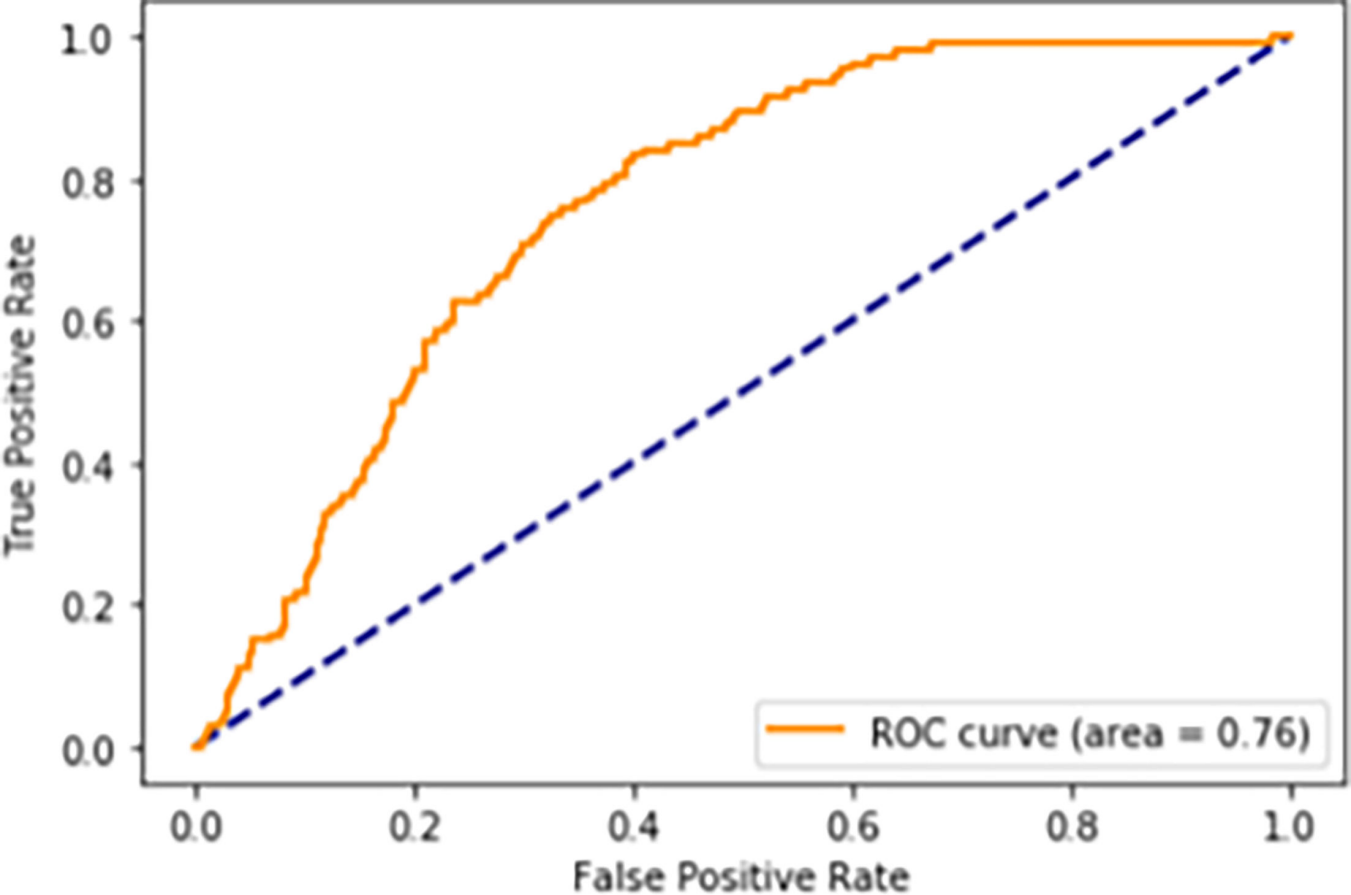
Receiver operating characteristic (ROC) curve for prediction of knee replacement on patient characteristics using support vector machine with seven parameters, missing target clinical performance.

The support vector machine (SVM) was the highest performing algorithm for predicting knee arthroplasty based only on patient characteristics (AUROC: 0.76) (Figure 1), but precision was low (AUPRC: 0.36) (Figure 1). Random forest, logistic regression, and XGBOOST all achieved similar but slightly lower performance with 0.74 AUROC (Table II). The inclusion of KL results significantly improved the model’s performance, raising performance of the AUROC from 0.76 to 0.88. However, to maintain independence from expert medical assessments, the KL grade was excluded from the final integrated model, with unannotated images analyzed directly instead.

**Table II.** Performance of different classifiers in knee arthroplasty prediction.

Model	SVM	Random forest	Gradient descent	Decision tree	Logistic regression	XGBOOST
AUROC	0.76	0.74	0.70	0.66	0.74	0.74

AUROC, area under the receiver operating characteristic curve; SVM, support vector machine.

### Predictions on radiographs

**Fig. 2:**
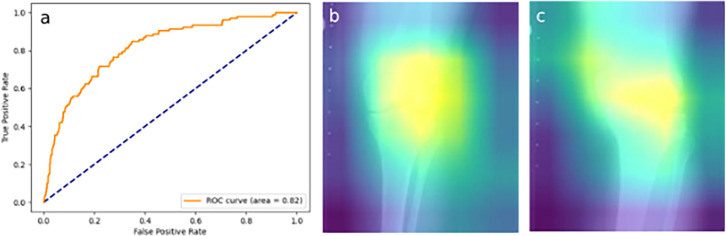
a) Receiver operating characteristic (ROC) curve for the DenseNet-201 architecture on Multicentre Osteoarthritis Study testing data; b) activation map for a high Kellgren-Lawrence (KL) knee with strong focus on a single lateral compartment; and c) activation map for a low KL knee with more diffuse focus.

In an initial transfer learning study (Table III), DenseNet-201 was the best convolutional neural network architecture at predicting knee arthroplasty on the radiographs (0.71 AUROC on MOST testing data), indicating superior predictive capability compared with ResNet-50, EfficientNetB0, and InceptionV2, which scored 0.57, 0.58, and 0.54, respectively. Hence, DenseNet-201 was selected for inclusion in the final architecture. After fine-tuning the algorithm, an AUROC of 0.82 and an AUPRC of 0.57 was achieved (Figure 2a). Explainability analyses using Grad-CAM highlighted the knee joint regions as the primary focus areas (Figures 2b and 2c), enhancing the model’s interpretability in identifying relevant anatomical features associated with OA.

**Table III.** Performance of different convolutional neural network architectures in an initial transfer learning study for knee arthroplasty prediction.

Architecture	DenseNet-201	ResNet-50	EfficientNetB0	InceptionV2
AUROC	0.71	0.57	0.58	0.54

AUROC, area under the receiver operating characteristic curve.

### Combined model and external validation

**Fig. 3:**
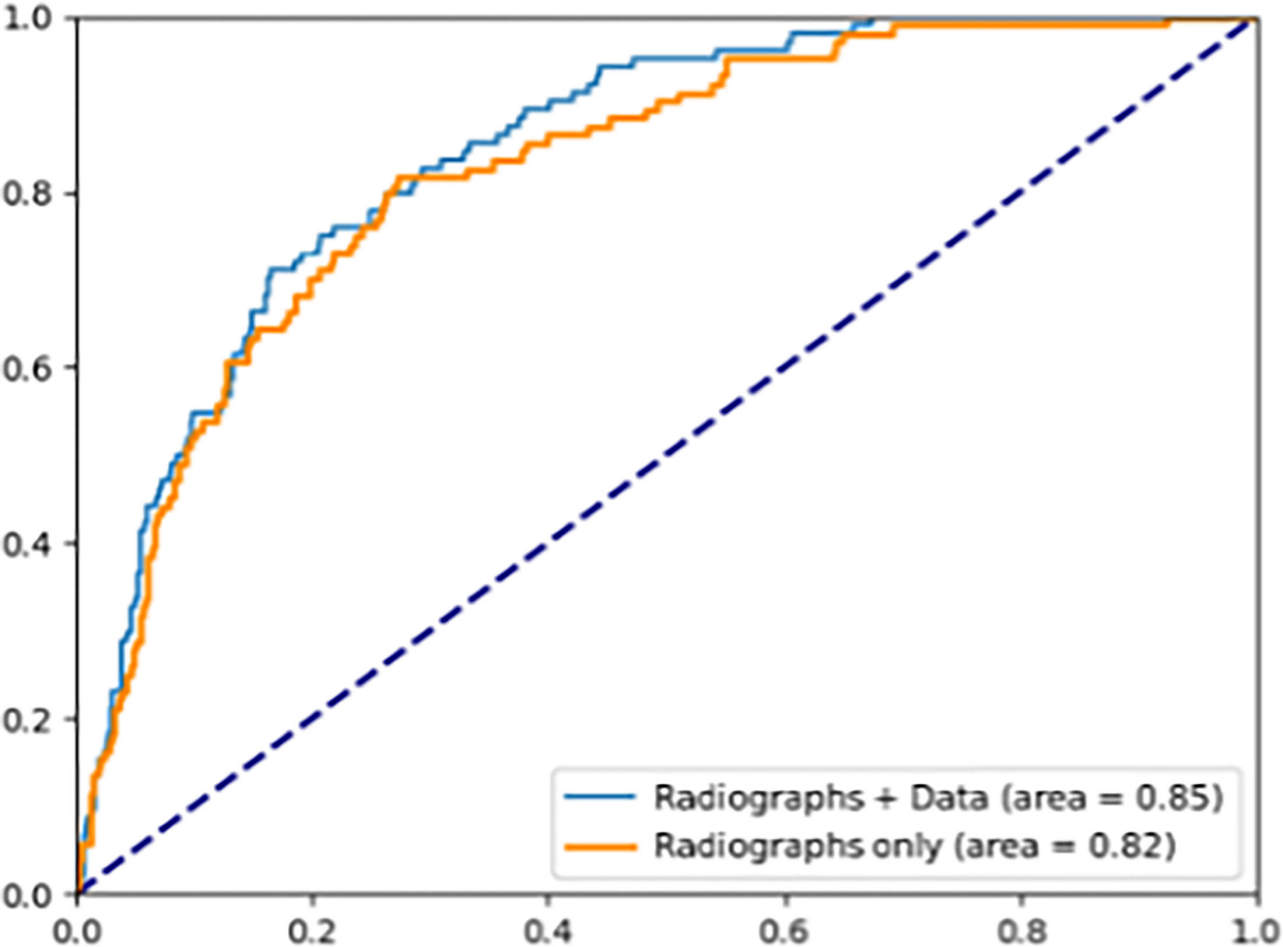
Receiver operating characteristic curve showing model performance after integrating both patient characteristics and imaging on the Multicentre Osteoarthritis Study test dataset.

Incorporating patient characteristics into the DenseNet-201 architecture improved performance of the algorithm to an AUROC of 0.85 and an AUPRC of 0.62 (Figure 3a), an improvement compared with the algorithms using only radiographs (AUROC: 0.82) or patient characteristics (AUROC: 0.76). The OAI database was used for external validation of the final model: the AUROC of 0.79 obtained on the OAI dataset was slightly lower than performance on the MOST testing dataset, but still indicated robust performance.

### Time to surgery

**Fig. 4:**
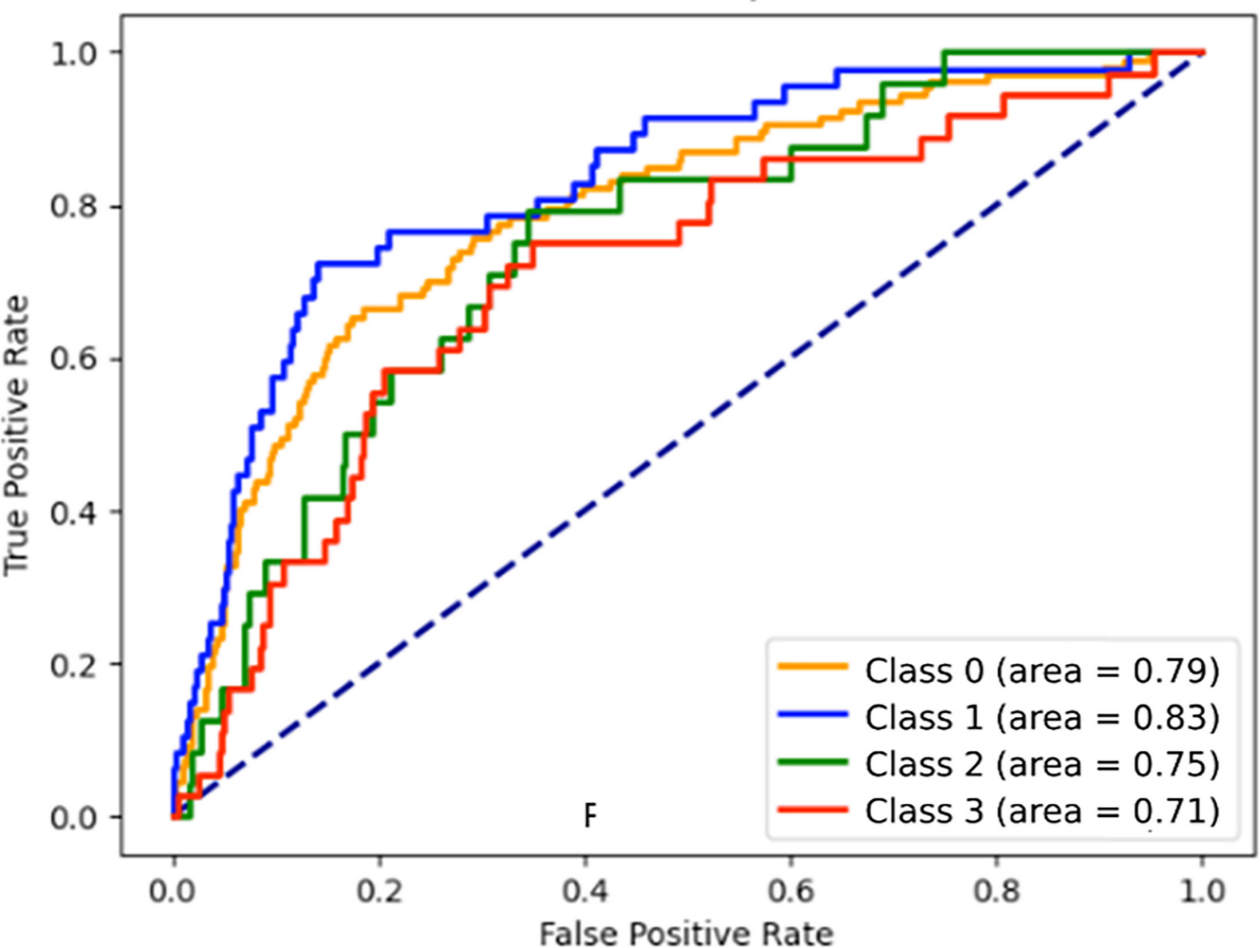
Receiver operating characteristic curves for surgical intervention within 40 months (Class 1), 40 to 70 months (Class 2), after 70 months (Class 3), or not all (Class 0), using the Osteoarthritis Initiative validation dataset.

The model demonstrated higher discriminatory performance for patients requiring earlier intervention (Figure 4) reflected by higher AUROC scores for patients requiring surgical interventions within 40 months (AUROC: 0.83) compared with patients undergoing surgery between 40 and 70 months (AUROC: 0.75) or after 70 months (AUROC: 0.71). Both the macro-average and weighted-average AUROC for forecasting the time to surgery were 0.76 across all classes. The model achieved AUPRC of 0.97 for patients not undergoing surgery, the majority cohort (prevalence: 0.90), whereas precision progressively decreased for patients undergoing surgery within 40 months (AUPRC: 0.26), between 40 and 70 months (AUPRC: 0.14), and after 70 months (AUPRC: 0.12).

## Discussion

In this study, we developed and validated a machine-learning model to predict the need for knee arthroplasty in patients with knee OA, using knee radiographs and various clinical data parameters as inputs. Tested on the internal dataset, our combined model achieved an AUROC of 0.85 and an AUPRC of 0.62 for knee arthroplasty at any point, outperforming models that used only one data source without using a large number of parameters (Tables II and III). Trained and tested on the MOST dataset, the model’s robustness was externally validated using the OAI database, with an overall AUROC of 0.79. The model also achieved well above the 2.7% prevalence baseline patients requiring surgery within 40 months (AUROC: 0.83; AUPRC: 0.26). This 40-month timepoint is of most relevance to hospital planning, as it relates to a timeframe over which resources can be planned locally.

While the SVM model incorporating KL grade achieved a higher AUROC (0.88) than the final integrated model (AUROC: 0.85), KL grade was deliberately excluded to enable development of a fully automated system that directly analyzes unannotated images, independent of expert medical assessment. Such reliance on expert-derived systems such as KL grading limits scalability and introduces interobserver variability. In this context, despite a slight reduction in performance, our integrated model offers greater potential in reproducibility and generalizability. Furthermore, the model is intended to complement clinical decision-making, rather than replace it, and the exclusion of KL grading does not preclude its integration into the overall clinical assessment.

Our findings align with and extend the current literature on predictive modelling in knee OA. Prior studies have shown the effectiveness of machine-learning algorithms in predicting total knee arthroplasty (TKA) using either clinical or imaging data, independently.^8,13^ Cigdem et al^27^ validated machine-learning models predicting TKA at two and five years using clinical and radiological data, achieving AUROCs of 0.913 and 0.873. Their approach excluded costly MRI, emphasizing scalability and clinical practicality. Whereas their study used interpretable models and externally validated performance, our work integrates multimodal data and achieves comparable predictive accuracy (AUROC 0.85) with added temporal granularity (e.g. prediction within 40 months).^27^ Jamshidi et al^28^ developed a machine-learning survival prediction model for TKA using the OAI dataset. Their DeepSurv-based model identified bone marrow lesions, KL grade, and knee-related symptoms as the three most influential features, achieving an AUROC of 0.86 and accurately predicting both the risk of TKA and the time to the event. While their study incorporated over 1,100 features, including MRI-based data, its reliance on costly and less routinely available imaging limits clinical scalability. In contrast, our model achieves comparable predictive accuracy (AUROC 0.85) without requiring MRI, instead integrating multimodal data derived from clinical and radiological inputs. Additionally, our use of Grad-CAM enhances interpretability of the imaging branch of our model, providing visual explanations for predictions, which Jamshidi et al’s^28^ DeepSurv model lacks, making our approach more practical and explainable for clinical application. However, it should be noted that Grad-CAM does not highlight specific pathological features in the knee or validate the model’s clinical reasoning.

Several unique aspects of our study distinguish it from prior research. First, the integration of DenseNet-201 architecture with routinely collected clinical data represents a novel approach, leveraging the strengths of deep learning for image analysis while incorporating traditional clinical predictors. Second, our model’s ability to predict the timing of surgical intervention adds a valuable dimension to predictive analytics in knee OA, offering clinicians a tool for better surgical planning. The model was better at predicting patients who would benefit from earlier intervention (surgical interventions within 40 months (AUROC: 0.83) vs patients undergoing surgery after 70 months (AUROC: 0.71)). This is an intuitive finding given that symptoms and objective radiological changes will be subtle at an earlier stage and their progression less predictable as lifestyle changes/events may impact progression over longer times. Third, the use of extensive external validation with the OAI dataset enhances the generalizability of our findings, addressing a common limitation in machine-learning studies that often train, test, and validate on single-cohort data.

This study has several limitations. The MOST and OAI are both observational cohorts rather than referral-based surgical populations, which could introduce a bias towards nonoperative patients, depressing precision-based metrics compared with what may be achieved in practice. Variability in imaging protocols and clinical practices across different institutions could affect the model’s performance in diverse settings. Additionally, the datasets used are both from the USA, which means that the algorithm needs to be validated using datasets from elsewhere in the world where the patient population and treatment approach might differ. Regarding the time-to-surgery analysis, conversion of follow-up time into coarse intervals may have resulted in a loss of temporal information and statistical power, and does not explicitly account for censoring, representing a limitation of this study. Another possible limitation of our study is the inclusion of TKA and unicompartmental knee arthroplasty (UKA) into a single primary outcome measure, as the indications, patient populations, and prognoses for these procedures differ. However, while there are differences between the nature of both procedures, the primary aim of our model was to predict progression to knee arthroplasty following failure of conservative management in knee OA patients, rather than guiding procedure selection. Nonetheless, future research incorporating procedure-specific outcomes may enable more accurate risk stratification for TKA and UKA individually, thereby enhancing clinical utility for surgical planning. Furthermore, while our implementation of Grad-CAM provided valuable, qualitative insight, it does not identify specific pathological features in the knee or validate the model’s clinical reasoning. Additionally, Grad-CAM produces low-spatial resolution activation maps that are typically coarse and may be unable to localize fine structural changes associated with knee OA, limiting its clinical utility. Lastly, although the selected clinical data were based on relevant and commonly collected parameters, integration of feature permutation importance would have improved transparency and clinical interpretability.

Despite these limitations, this study has several significant strengths. The large sample size and comprehensive data from the MOST and OAI databases provide a robust foundation for model development and validation. The application of advanced machine-learning techniques, including transfer learning with DenseNet-201, enhances the model’s predictive capability. Additionally, external validation with an independent dataset confirms the model’s generalizability, which is crucial for clinical application.

In conclusion, this study highlights the potential of deep learning models, which integrate clinical and radiological data, to predict the need for knee arthroplasty. The robust performance and generalizability of the developed model could streamline clinical pathways and predict local demand for surgery during the next three years. This will facilitate resource planning for providers and accurate and timely access to surgical interventions for patients. Future research should focus on refining temporal predictions and further validating the model across diverse clinical settings. Additionally, investigating the sources of external validation performance drop should be considered in future research to better characterize its decline and identify potential sources of bias.

**Take home message**

- This study developed and validated a deep learning model integrating routine clinical data and knee radiographs, achieving high predictive accuracy (area under the receiver operating characteristic curve (AUROC) of 0.85) for the need for knee arthroplasty in patients with knee osteoarthritis.

- External validation using the Osteoarthritis Initiative dataset confirmed the model’s robustness and generalizability, with an AUROC of 0.79.

- The timing prediction model performed especially well for surgery within 40 months (AUROC 0.83) demonstrating its potential utility in clinical decision-making and resource allocation within this timeframe.

## Data Availability

The datasets generated and analyzed in the current study are not publicly available due to data protection regulations. Access to data is limited to the researchers who have obtained permission for data processing. Further inquiries can be made to the corresponding author.
